# Viral clearance after early corticosteroid treatment in patients with moderate or severe covid-19

**DOI:** 10.1038/s41598-020-78039-1

**Published:** 2020-12-04

**Authors:** V. Spagnuolo, M. Guffanti, L. Galli, A. Poli, P. Rovere Querini, M. Ripa, M. Clementi, P. Scarpellini, A. Lazzarin, M. Tresoldi, L. Dagna, A. Zangrillo, F. Ciceri, A. Castagna, COVID-BioB study group, A. Andolina, M. Baiardo Redaelli, E. Baldissera, G. Bigai, A. Bigoloni, N. Boffini, G. Borio, S. Bossolasco, E. Bruzzesi, M. G. Calabrò, S. Calvisi, C. Campochiaro, D. Canetti, V. Canti, J. Castellani, B. Castiglioni, G. Cavalli, L. Cavallo, M. Cernuschi, M. Chiurlo, M. Cilla, E. Cinel, P. Cinque, C. Conte, V. Da Prat, A. Danise, R. De Lorenzo, G. De Luca, A. Dell’Acqua, R. Dell’Acqua, E. Della-Torre, L. Della Torre, G. Di Terlizzi, I. Dumea, F. Farolfi, M. Ferrante, C. Frangi, L. Fumagalli, G. Gallina, B. Germinario, N. Gianotti, H. Hasson, F. Lalla, G. Landoni, M. Lanzillotta, R. Li Voti, A. Mastrangelo, E. Messina, E. Moizo, M. Montagna, G. Monti, G. Morsica, C. Muccini, S. Nozza, C. Oltolini, M. Pascali, A. Patrizi, M. Pieri, D. Prestifilippo, G. Ramirez, M. Ranzenigo, J. Sapienza, S. Sartorelli, F. Seghi, G. Tambussi, C. Tassan Din, A. Tomelleri, S. Turi, C. Uberti-Foppa, C. Vinci

**Affiliations:** 1 Unit of Infectious Diseases, Istituto di Ricovero e Cura a Carattere Scientifico (IRCCS), San Raffaele Scientific Institute, Milan, Italy; 2 Vita-Salute San Raffaele University, Milan, Italy; 3 Internal Medicine, Diabetes, and Endocrinology Unit, IRCCS San Raffaele Scientific Institute, Milan, Italy; 4 Unit of Microbiology and Virology, IRCCS San Raffaele Scientific Institute, Milan, Italy; 5 General Medicine and Advanced Care Unit, IRCCS San Raffaele Scientific Institute, Milan, Italy; 6 Unit of Immunology, Rheumatology, Allergy and Rare Diseases, IRCCS San Raffaele Scientific Institute, Milan, Italy; 7 Anesthesia and Intensive Care Department, IRCCS San Raffaele Scientific Institute, Milan, Italy; 8 Hematology and Bone Marrow Transplant Unit, IRCCS San Raffaele Scientific Institute, Milan, Italy

**Keywords:** Diseases, Infectious diseases, Viral infection

## Abstract

The aim of this study was to evaluate the impact of early treatment with corticosteroids on SARS-CoV-2 clearance in hospitalized COVID-19 patients. Retrospective analysis on patients admitted to the San Raffaele Hospital (Milan, Italy) with moderate/severe COVID-19 and availability of at least two nasopharyngeal swabs. The primary outcome was the time to nasopharyngeal swab negativization. A multivariable Cox model was fitted to determine factors associated with nasopharyngeal swab negativization. Of 280 patients included, 59 (21.1%) patients were treated with steroids. Differences observed between steroid users and non-users included the proportion of patients with a baseline PaO_2_/FiO_2_ ≤ 200 mmHg (45.8% vs 34.4% in steroids and non-steroids users, respectively; p = 0.023) or ≤ 100 mmHg (16.9% vs 12.7%; p = 0.027), and length of hospitalization (20 vs 14 days; p < 0.001). Time to negativization of nasopharyngeal swabs was similar in steroid and non-steroid users (p = 0.985). According to multivariate analysis, SARS-CoV-2 clearance was associated with age ≤ 70 years, a shorter duration of symptoms at admission, a baseline PaO_2_/FiO_2_ > 200 mmHg, and a lymphocyte count at admission > 1.0 × 10^9^/L. SARS-CoV-2 clearance was not associated with corticosteroid use. Our study shows that delayed SARS-CoV-2 clearance in moderate/severe COVID-19 is associated with older age and a more severe disease, but not with an early use of corticosteroids.

## Introduction

As of October 26 2020, the ongoing pandemic of severe acute respiratory syndrome-Coronavirus 2 (SARS-CoV-2) has caused more than 43 million cases of Coronavirus disease-19 (COVID-19), resulting in more than 1,150,000 deaths^1^. Severe forms of COVID-19 are typically characterized by bilateral interstitial pneumonia and hyperactivation of the inflammatory cascade^2^. Considering the current lack of proven antiviral therapy, several different immunosuppressive agents have been evaluated with the aim of reducing the hyperinflammatory status associated with COVID-19 and improving the patients’ prognosis^3^.

Corticosteroids are inexpensive and readily available agents that are widely used for their anti-inflammatory effects in patients with respiratory infections. Earlier studies indicated that the use of corticosteroids in patients with SARS and MERS was associated with delayed viral clearance, and no clear benefits in term of survival, length of hospitalization, or use of mechanical ventilation^4^.

For these reasons, in the initial phase of SARS-CoV-2 pandemic, use of corticosteroids for the management of COVID-19 patients was not recommended. However, findings from non-randomized studies demonstrated a lower mortality in patients with COVID-19 when treated with corticosteroids^5, 6^. These preliminary findings were confirmed by a recent prospective meta-analysis^7^, including 7 randomized clinical trials^7–12^ that showed a clear survival benefit in critically ill patients with COVID-19 who received corticosteroids.

According to these findings, World Health Organization (WHO) now recommend systemic corticosteroids for the treatment of patients with severe and critical COVID-19^13^.

However, very little data on SARS-CoV-2 viral clearance after steroid treatment is currently available^14, 15^. The aim of this study was to evaluate the impact of an early treatment with corticosteroids on SARS-CoV-2 viral clearance in hospitalized COVID-19 patients.

## Results

Two-hundred and eighty patients were included in this study. The median age was 63.5 (53.5–74.0) years, 34% of patients were > 70 years, 78% were males, 92% Caucasian, 3% were active smokers, 74% were overweight, and 66% had at least one comorbidity (including diabetes (18%), hypertension (45%), and any cardiovascular (29%), neoplastic (15%) or respiratory (9%) disease). At hospital admission, COVID-19 associated symptoms had been present for 7 days (4–10), while 36.8% and 13.6% of patients had a PaO_2_/FiO_2_ ≤ 200 mmHg or ≤ 100 mmHg, respectively. Serum levels of C-reactive Protein (CRP), lactate dehydrogenase (LDH) and ferritin were 70.9 (28.2–121.6) mg/L, 340 (275–449) U/L, and 1068 (561–1876) ng/mL, respectively; plasma D-dimer levels were 1.01 (0.59–2.05) µg/mL, total lymphocytes were 1.0 (0.8–1.3) × 10^9^/L, and the neutrophils/lymphocytes ratio was 5.0 (3.1–8.6). Baseline characteristics of patients according to steroid use are reported in Table 1.

**Table 1:** Patients’ characteristics at the time of hospitalization for a moderate or severe COVID-19 infection.

Characteristics	Overall (n = 280)	Use of steroid (n = 59)	No use of steroid (n = 221)	p-value^§^
Age (years)	63.5 (53.5–74)	67 (54–77)	62 (53–73)	0.161
Male gender	217 (77.5%)	46 (78%)	171 (77.4%)	0.999
**Ethnicity**				0.503
White	255 (91.7%)	54 (91.5%)	201 (91.8%)	
Latin	19 (6.8%)	5 (8.5%)	14 (6.4%)	
Other	6 (1.4%)	0 (0%)	6 (1.8%)	
Smoke (active or ex)	36 (12.9%)	11 (18.6%)	25 (11.3%)	0.187
Fever (°C)	37.8 (36.8–38.3)	37.9 (37–38.3)	37.7 (36.6–38.3)	0.287
Systolic blood pressure (mmHg)	127 (115–140)	125 (120–140)	130 (115–140)	0.728
Diastolic blood pressure (mmHg)	75 (70–80)	72 (70–80)	75 (70–80)	0.332
**Body mass index (BMI, kg/m**^**2**^**)**				0.291
≤ 25	62 (26.4%)	8 (17.4%)	54 (28.6%)	
> 25–30	115 (48.9%)	26 (56.5%)	89 (47.1%)	
> 30	58 (24.7%)	12 (26.1%)	46 (24.3%)	
**Number of comorbidities**				0.599
0	94 (33.6%)	16 (27.1%)	78 (35.3%)	
1	81 (28.9%)	18 (30.5%)	63 (28.5%)	
2	56 (20%)	12 (20.3%)	44 (19.9%)	
3	49 (17.5%)	13 (22%)	36 (16.3%)	
Cardiovascular disease	81 (28.9%)	20 (33.9%)	61 (27.6%)	0.338
Diabetes	49 (17.5%)	12 (20.3%)	37 (16.7%)	0.563
Hypertension	126 (45%)	26 (44.1%)	100 (45.2%)	0.859
Malignancies	43 (15.4%)	12 (20.3%)	31 (14%)	0.229
Asthma	8 (2.9%)	4 (6.8%)	4 (1.8%)	0.111
Chronic obstructive pulmonary disease	16 (5.7%)	6 (10.2%)	10 (4.5%)	0.077
Oxygen saturation (%)	94 (92–97)	94 (91–97)	95 (92–96)	0.901
**PO**_**2**_**/FiO**_**2**_				0.023
> 200	131 (46.8%)	29 (49.2%)	102 (46.2%)	
≤ 200	103 (36.8%)	27 (45.8%)	76 (34.4%)	
Unknown	46 (16.4%)	3 (5.1%)	43 (19.5%)	
**PO**_**2**_**/FiO**_**2**_				0.027
> 100	196 (70%)	46 (78%)	150 (67.9%)	
≤ 100	38 (13.6%)	10 (16.9%)	28 (12.7%)	
Unknown	46 (16.4%)	3 (5.1%)	43 (19.5%)	
Days from symptoms to hospital admission	7 (4–10)	7.5 (4.5–10)	7 (4–10)	0.859
Lactate dehydrogenase (U/L)	340 (275–449)	329 (271–453)	342 (275–447)	0.782
Normal range: [125–220]	N = 269	N = 59	N = 210	
White Blood Cells (10^9^cells/L)	6.8 (5.2–9)	7.2 (5–10.1)	6.8 (5.2–8.6)	0.429
Normal range: [4.8–10.8]	N = 269	N = 57	N = 212	
Total lymphocytes (10^9^cells/L)	1 (0.8–1.3)	0.9 (0.6–1.5)	1 (0.8–1.3)	0.603
Normal range: [1.0–4.8]	N = 275	N = 59	N = 216	
Glucose (mg/dL)	108 (99–126)	109.5 (97–134)	108 (99–125)	0.747
Normal range: [60–100]	N = 273	N = 59	N = 214	
D-Dimer (µg/mL)	1.01 (0.59–2.05)	1.09 (0.49–2.09)	0.99 (0.59–2.02)	0.649
Normal range: [0.27–0.77]	N = 140	N = 35	N = 105	
C-reactive protein (mg/L)	71 (28–122)	90 (48–129)	68 (26–120.4)	0.052
Normal range: [0–6]	N = 276	N = 58	N = 218	
Ferritin (ng/mL)	1068 (561–1876)	840 (392–1576)	1076 (608–2009)	0.116
Normal range: [male:30–400; female: 15–150]	N = 166	N = 39	N = 127	
Fibrinogen (mg/dL)	582 (481–675)	639 (593–714.5)	561 (462–656)	0.013
Normal range: [150–400]	N = 106	N = 24	N = 82	
N terminal pro B type natriuretic peptide (pg/mL)	191 (73–545)	213 (77–1088)	186 (67–505)	0.230
Normal range: [male ≤ 50 years: < 89; male > 50 years: < 228; female ≤ 50 years: < 154; female > 50 years: < 335]	N = 134	N = 30	N = 104	
Troponin (ng/mL)	10.7 (6.2–20.8)	12.5 (6.9–26.1)	10.2 (5.8–20.2)	0.267
Normal range: [0–14]	N = 163	N = 36	N = 127	

Results are described by median (IQR) or frequency (%).

^§^By the Wilcoxon rank-sum test or the chi-square/Fisher’s exact test, as appropriate.

During hospitalization, 12 (4.3%) patients died, 24 (8.6%) were admitted to the intensive care unit (ICU), and 95 (34%) required mechanical ventilation (invasive or non-invasive). Antiretrovirals, hydroxychloroquine, and immunomodulatory agents were administered to 222 (79%), 261 (93%), and 97 (35%) patients, respectively.

Fifty-nine (21.1%) patients were treated with steroids, after a median of 1 day (0–2) since admission, and for a total of 9 (7–16) days. Initially, intravenous methylprednisolone was used in 55 (93.2%) cases, oral prednisone in 3 (5.1%) cases, and intravenous dexamethasone in 1 (1.7%) case of steroid use. Initial steroid methylprednisolone-equivalent dosage was 0.87 (0–51 to 1.0) mg/Kg.

At steroid discontinuation, 44 (74.6%) steroid users were receiving intravenous methylprednisolone, 10 (16.9%) oral prednisone, and 5 (8.5%) intravenous dexamethasone. Methylprednisolone-equivalent dosage was 0.38 (0.21–0.53) mg/Kg.

Differences between steroid users and non-users were observed with regard to the proportion of patients with a baseline PaO_2_/FiO_2_ ≤ 200 mmHg (45.8% vs 34.4% in steroids and non-steroids users, respectively; p = 0.023) and ≤ 100 mmHg (16.9% vs 12.7%, p = 0.027), and with regard to the length of hospitalization (20 vs 14 days; p < 0.001). Although steroid users had a higher proportion of severe respiratory impairment at admission than non-users, no significant differences between the two groups were found with regard to mortality (6.8% vs 3.6%; p = 0.29), use of mechanical ventilation (36% vs 34%; p = 0.76), and risk of subsequent infections (10.4% in both groups; p = 0.87). Among 47/59 steroid users without a previous diabetes mellitus diagnosis, 2 (4.3%) steroid users developed new-onset diabetes during hospitalization.

**Figure 1:**
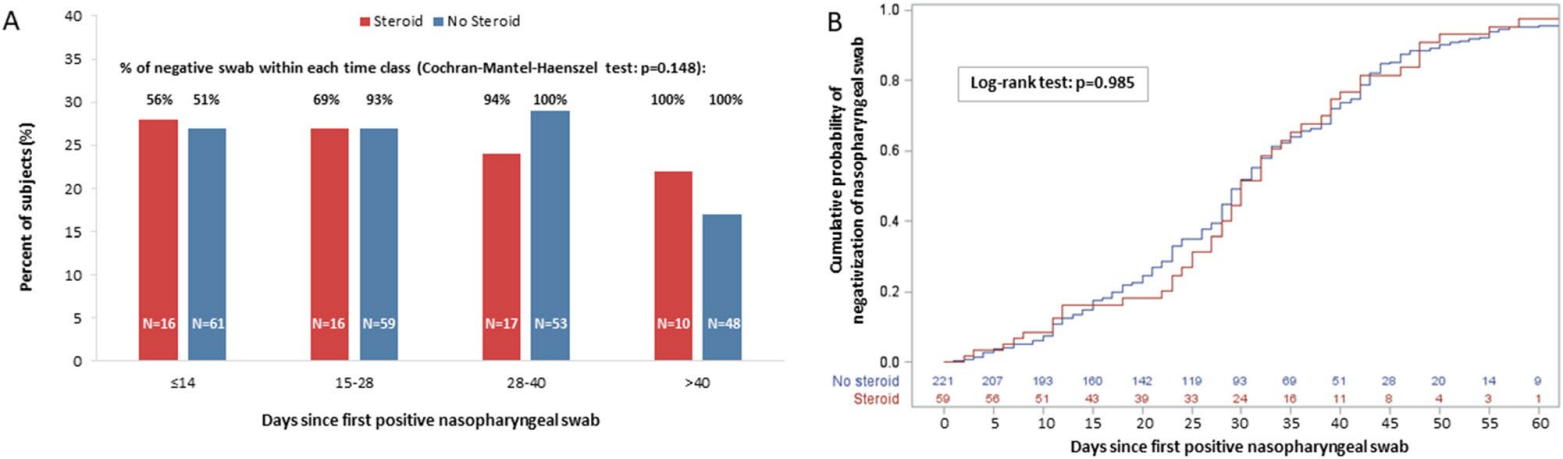
Distribution of follow-up nasopharyngeal swabs according to days since first positive swab and use of steroid (**A**); time to negativization of nasopharyngeal swab according to the use of steroid (**B**).

**Figure 2:**
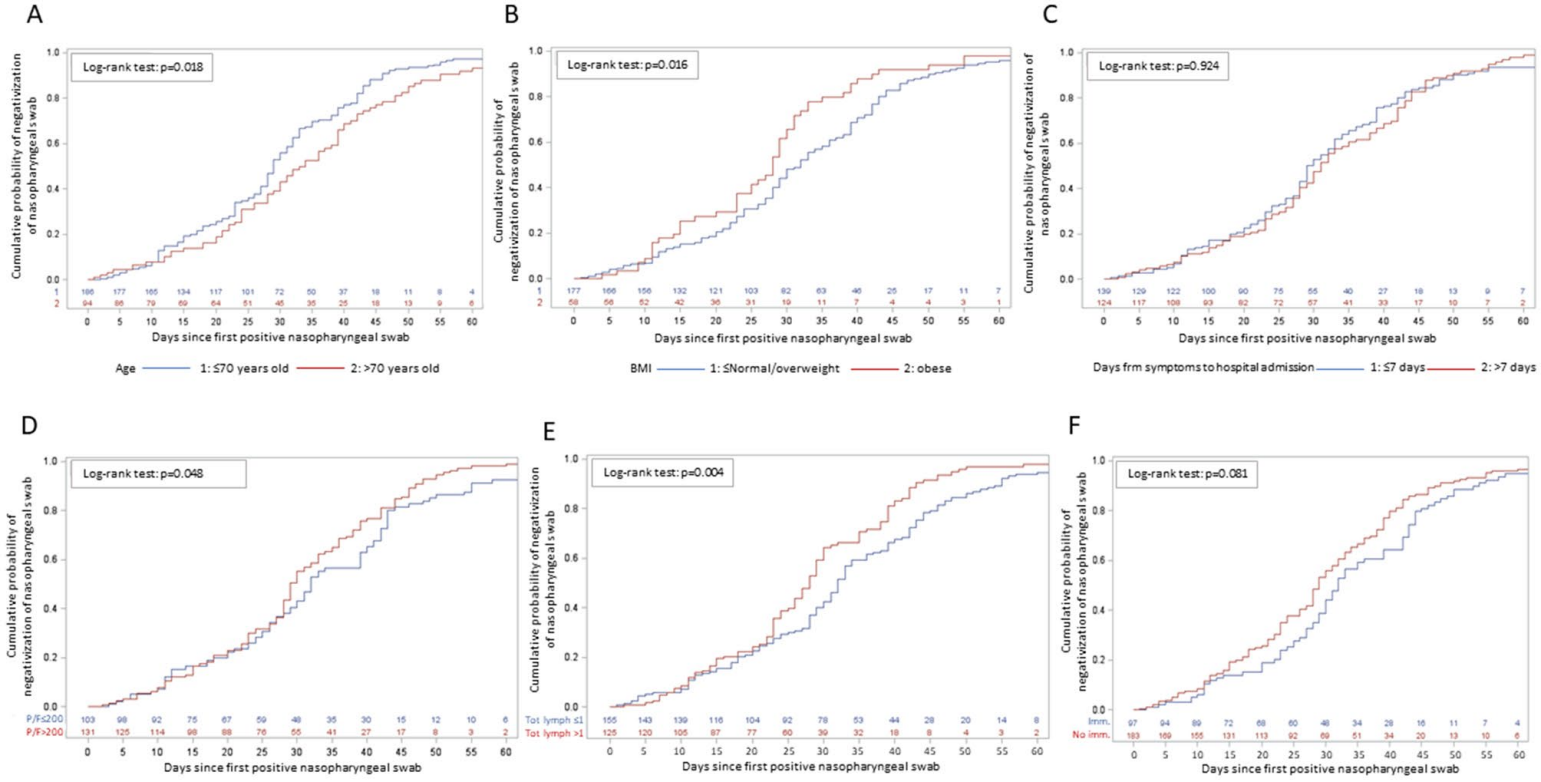
Time to negativization of nasopharyngeal swab according to: age (**A**); body mass index (**B**); days from symptoms to hospital admission (**C**); PaO_2_/FiO_2_ at hospital admission (**D**); total lymphocytes count at hospital admission (**E**); use of immunomodulatory drugs (**F**).

During follow-up, each patient underwent 4 (3–5) consecutive nasopharyngeal swabs. The distribution of follow-up nasopharyngeal swabs, and the proportion of negative samples according to days since first positive swab and use of steroids are reported in Fig. 1. Time to negativization of nasopharyngeal swabs according to the use of steroids, immunomodulatory agents, baseline lymphocyte cell count, PaO_2_/FiO_2_, age and days from symptoms occurrence to hospital admission is also shown in Figs. 1 and 2.

Using multivariate analysis (Table 2), SARS-CoV-2 clearance was associated with age ≤ 70 years (aHR = 1.57, CI 1.11–2.23; p = 0.011), shorter symptoms duration at hospital admission (aHR for 7-days longer = 0.76 (0.61–0.94); p = 0.013), baseline PaO2/FiO2 > 200 mmHg (aHR = 1.42, CI 1.03–1.97; p = 0.035), and a lymphocyte count at admission > 1.0 × 109/L (aHR = 1.55, CI 1.12–2.15; p = 0.009). Use of corticosteroids did not impact on viral clearance (p = 0.162).

**Table 2:** Multivariable analysis (stepwise Cox proportional hazard model): factors associated with the risk of negativization of nasopharyngeal swab.

Covariates	Category	Adjusted hazard ratio (95% confidence interval)	p-value
Age, years	≤ 70 vs > 70	1.57 (1.11–2.23)	0.011
Days from symptoms to hospital admission	Per 5-days longer	0.76 (0.61–0.94)	0.013
PaO_2_/FiO_2_	> 200 vs ≤ 200	1.42 (1.03–1.97)	0.035
Total lymphocytes, per 10^9^/L	> 1 vs ≤ 1	1.55 (1.12–2.15)	0.009

All covariates were measured at baseline. The other tested variables were: gender, body mass index (normal/overweight vs obese), the number of comorbidities* (≥ 1 vs none), use of immunomodulatory drugs (yes vs no), use of antiviral drugs or hydroxychloroquine (yes vs no), use of steroid (yes vs no), lactate dehydrogenase (≤ 330 vs > 330), C-reactive protein (≤ 68.7 vs > 68.7).

*The following comorbidities were considered: malignancies, diabetes, cardiovascular disease, hypertension, asthma, chronic obstructive pulmonary disease.

## Discussion

A concern against the use of corticosteroids in COVID-19 is the potential negative impact of steroids on the control of SARS-CoV-2 viral replication and the consequent delayed viral clearance, as reported in other viral pneumonia^4^. Our study shows that steroid treatment has no impact on viral clearance in patients with moderate or severe COVID-19. Our results are similar to those reported in two other studies on smaller cohorts of patients^14, 15^.

In our study, we also observed an association between delayed viral clearance and older age. The characteristic age-related immune decline observed in elderly patients^16^ may impair their ability to control SARS-CoV-2 infection, potentially explaining the higher risk of viral persistence observed in subjects ≥ 70 years. A delayed viral clearance was also related with a longer duration of symptoms before hospitalization and with respiratory impairment and lymphopenia at admission. All these different factors may reflect a more severe disease and consequently a higher probable viral load in the respiratory tract^17^.

Moreover, we observed a longer length of hospitalization in steroid-users compared to non-users. We think that this difference may be explained by the higher degree of baseline respiratory impairment, as documented by the higher proportion of patients with PaO_2_/FiO_2_ ≤ 200 and ≤ 100, in steroid users.

The pathophysiological mechanism associated with the development of progressive lung damage associated with respiratory failure in COVID-19 is related to an unregulated pro-inflammatory cytokine response^18^.

At this regard, the steroid anti-inflammatory effect plays a role in mitigating the hyper-inflammatory status that characterizes the disease progression. Currently, randomized clinical trials^7–12^ demonstrated the benefit in overall survival in critically-ill patients with COVID-19 accordingly to this biological mechanism.

Potential concerns regarding the use of steroids in COVID-19 management include side-effects, in particular metabolic disorders and secondary infections. However, in patients with severe/critical illness, the benefit of steroid administration overcome the potential risks.

Our study has several limitations. First, we cannot exclude a potential selection bias, given that the included patients need to have at least two nasopharyngeal swabs. This criterion excluded patients showing a more aggressive course who unfortunately died within a few days of hospital admission (leading also to an underestimation of the number of observed deaths). Second, treatment with steroids was not standardized, and the decision to administer this drug and the timing of administration was at the discretion of the different physicians. This approach might be associated with a potential indication bias.

Third, we had not available data on baseline SARS-CoV-2 viral load (cycle threshold (Ct) values) that might confound the association between corticosteroid treatment and viral clearance.

In conclusion, our study showed that delayed SARS-CoV-2 clearance in moderate/severe COVID-19 was associated with older age and a more severe disease, but not with an early use of corticosteroids. Considering the growing body of scientific evidences^5–12^ on steroid efficacy in improving survival in COVID-19 patients, our findings may reassure clinicians on the concern of a potential delayed viral clearance.

## Methods

For this retrospective analysis, we considered all patients admitted between February 25th 2020 and May 19th 2020 to the Istituto di Ricovero e Cura a Carattere Scientifico (IRCCS) San Raffaele (Milan, Italy) with moderate or severe COVID-19, a definite outcome (discharge or death), complete information on therapies administered during hospitalization, and the availability of at least two nasopharyngeal swabs (one at hospital admission and ≥ 1 thereafter).

We obtained data from the COVID-BioB clinical database of the IRCCS San Raffaele Hospital. The study was approved by the Ethics Committee of San Raffaele Hospital (protocol No. 34/int/2020) and was registered on ClinicalTrials.gov (NCT04318366). All patients signed an informed consent form. Our research was in compliance to the Declaration of Helsinki.

COVID-19 was diagnosed in all patients with a SARS-CoV-2 positive real-time reverse-transcriptase polymerase chain reaction (RT-PCR; Roche Cobas Systems) assay result from a nasopharyngeal swab and compatible signs, symptoms, and/or radiological findings. All nasopharyngeal samples were submitted to the San Raffaele Scientific Institute Laboratory for RT-PCR testing, yielding qualitative results (positive or negative).

Moderate COVID-19 was defined as the presence, during hospitalization, of: (1) at least one arterial oxygen partial pressure (PaO_2_)/fraction inspired oxygen (FiO_2_) ratio < 300 mmHg, as determined by arterial blood gas analysis; or (2) supplemental oxygen use; or (3) a peripheral saturation of oxygen < 94%.

Severe COVID-19 was defined as: requiring the need of mechanical ventilation (both invasive and non-invasive). Only steroid treatment within 7 days of admission was considered for this analysis. Patients on chronic steroid therapy were excluded.

Use of corticosteroids in patients with COVID-19 was at the discretion of the different medical teams. All corticosteroids were converted to methylprednisolone-equivalent doses and dosing was reported in mg/Kg. Other treatments considered in the analysis included immunomodulatory agents (tocilizumab, sarilumab, mavrilimumab, and anakinra), hydroxychloroquine, and antiretrovirals (lopinavir/ritonavir and darunavir/cobicistat).

The primary outcome of this study was the time to nasopharyngeal swab negativization defined by: (i) the occurrence of two consecutive negative swabs after hospital admission (baseline), in cases of multiple nasopharyngeal swabs; or (ii) the occurrence of a negative swab prior to discharge or death, in cases without multiple nasopharyngeal swabs. In patients treated with corticosteroids, swab negativization (if shown) was attributed to corticosteroid treatment only if it had occurred after steroid introduction.

### Statistical analyses

Results were reported as median (interquartile range, IQR) and frequency (%).

Distributions of continuous variables were compared between patients treated with or not treated with steroids using the Wilcoxon rank-sum test or the chi-square/Fisher exact test for categorical variables.

Time to nasopharyngeal swab negativization was estimated by the use of Kaplan–Meier curves; estimates were provided according to different factors and compared by the log-rank test. Follow-up started at baseline and ended at the date of first nasopharyngeal swab negativization, or the date of discharge, or death (whichever occurred first), and was right censored 60 days after baseline owing to the low number of cases thereafter; there were no competing events.

A multivariable Cox proportional hazard model was fitted to determine factors associated with the risk of nasopharyngeal swab negativization; the adjusted hazard ratio (aHR) with the corresponding 95% CI were reported. The considered covariates were fitted as time-fixed and measured at baseline. A backward elimination variable selection algorithm with entry and stay criteria of 0.10 and 0.05, respectively, was applied; adjusted hazard ratios (aHR) of nasopharyngeal swab negativization were reported with the corresponding 95% CI for significant covariates. The assumption of the proportional hazard was examined by use of interactions of the predictors and the function of time; it was confirmed for all the significant covariates.

For the analyses, two-sided p-values < 0.05 were considered statistically significant. All analyses were performed using the SAS Software, release 9.4 (SAS Institute, Cary, NC).

## Data Availability

The datasets used and analysed during the current study are available from the corresponding author on reasonable request.
