# Publisher Correction: Viral clearance after early corticosteroid treatment in patients with moderate or severe covid-19

**DOI:** 10.1038/s41598-021-89270-9

**Published:** 2021-05-17

**Authors:** V. Spagnuolo, M. Guffanti, L. Galli, A. Poli, P. Rovere Querini, M. Ripa, M. Clementi, P. Scarpellini, A. Lazzarin, M. Tresoldi, L. Dagna, A. Zangrillo, F. Ciceri, A. Castagna, COVID-BioB study group, A. Andolina, M. Baiardo Redaelli, E. Baldissera, G. Bigai, A. Bigoloni, N. Boffini, G. Borio, S. Bossolasco, E. Bruzzesi, M. G. Calabrò, S. Calvisi, C. Campochiaro, D. Canetti, V. Canti, J. Castellani, B. Castiglioni, G. Cavalli, L. Cavallo, M. Cernuschi, M. Chiurlo, M. Cilla, E. Cinel, P. Cinque, C. Conte, V. Da Prat, A. Danise, R. De Lorenzo, G. De Luca, A. Dell’Acqua, R. Dell’Acqua, Emanuel Della-Torre, L. Della Torre, G. Di Terlizzi, I. Dumea, F. Farolfi, M. Ferrante, C. Frangi, L. Fumagalli, G. Gallina, B. Germinario, N. Gianotti, H. Hasson, F. Lalla, G. Landoni, M. Lanzillotta, R. Li Voti, A. Mastrangelo, E. Messina, E. Moizo, M. Montagna, G. Monti, G. Morsica, C. Muccini, S. Nozza, C. Oltolini, M. Pascali, A. Patrizi, M. Pieri, D. Prestifilippo, G. Ramirez, M. Ranzenigo, J. Sapienza, S. Sartorelli, F. Seghi, G. Tambussi, C. Tassan Din, A. Tomelleri, S. Turi, C. Uberti-Foppa, C. Vinci

**Affiliations:** 1 Unit of Infectious Diseases, Istituto di Ricovero e Cura a Carattere Scientifico (IRCCS), San Raffaele Scientific Institute, Milan, Italy; 2 Vita-Salute San Raffaele University, Milan, Italy; 3 Internal Medicine, Diabetes, and Endocrinology Unit, IRCCS San Raffaele Scientific Institute, Milan, Italy; 4 Unit of Microbiology and Virology, IRCCS San Raffaele Scientific Institute, Milan, Italy; 5 General Medicine and Advanced Care Unit, IRCCS San Raffaele Scientific Institute, Milan, Italy; 6 Unit of Immunology, Rheumatology, Allergy and Rare Diseases, IRCCS San Raffaele Scientific Institute, Milan, Italy; 7 Anesthesia and Intensive Care Department, IRCCS San Raffaele Scientific Institute, Milan, Italy; 8 Hematology and Bone Marrow Transplant Unit, IRCCS San Raffaele Scientific Institute, Milan, Italy

Correction to: *Scientific Reports* 10.1038/s41598-020-78039-1, published online 04 December 2020

In the original version of this Article, the author from a consortium Della Torre Emanuel was incorrectly indexed. This error has now been corrected.

